# Global, regional, and national consumption of controlled opioids: a cross-sectional study of 214 countries and non-metropolitan territories

**DOI:** 10.1177/20494637211013052

**Published:** 2021-05-04

**Authors:** Georgia C Richards, Jeffrey K Aronson, Kamal R Mahtani, Carl Heneghan

**Affiliations:** 1 Global Centre on Healthcare and Urbanisation, Kellogg College, University of Oxford, Oxford, UK; 2 Centre for Evidence-Based Medicine, Nuffield Department of Primary Care Health Sciences, University of Oxford, Oxford, UK

**Keywords:** Opioids, pain management, anaesthetics, analgesics, antidiarrheals, opioid substitution therapies, cough suppressants, narcotics

## Abstract

**Introduction:**

The consumption of opioids has increased globally since the 1990s. Previous studies of global opioid consumption have concentrated on morphine alone or a subset of opioids, with a focus on cancer pain and palliative care. In this study, we have determined the global, regional, and national consumption of all controlled opioids, including anaesthetics, analgesics, antidiarrheals, opioid substitution therapies, and cough suppressants.

**Methods:**

We conducted a cross-sectional study using data from the International Narcotics Control Board (INCB). We calculated mean opioid consumption (mg/person) globally, regionally, and nationally for 2015–2017, where consumption refers to the total amount of controlled opioids distributed for medical purposes and excludes recreational use. We ranked countries by total consumption and quantified the types of opioids consumed globally.

**Results:**

Between 2015 and 2017, 90% of the world’s population consumed only 11% of controlled opioids. An average of 32 mg/person was consumed annually, but this was not equally distributed across the world. Consumption was the highest in Germany (480 mg/person), followed by Iceland (428 mg/person), the United States (398 mg/person) and Canada (333 mg/person). Oxycodone (35%) was the most heavily consumed controlled opioid globally, followed by morphine (15.9%), methadone (15.8%) and tilidine (14%).

**Conclusion:**

Large disparities persist in most of the world in accessing essential opioid medicines. Consumption patterns should continue to be monitored, and collaborative strategies should be developed to promote access and the appropriate prescribing of opioids in all countries and non-metropolitan territories.

## Introduction

Access to medicines is widely recognized as a human right,^1^ although many countries experience barriers to access. The World Health Organization (WHO) considers five opioids to be essential medicines, including codeine, fentanyl, loperamide, methadone, and morphine, which are listed in its Model List of Essential Medicines.^2,3^ The WHO’s analgesic ladder for cancer pain and the recognition that pain relief is a patient and human right led to a global increase in opioid consumption during the 1990s,^4–6^ particularly in the Americas, Europe and Oceania, which account for most of the consumption.^7–10^ But this growth in opioid consumption was less pronounced in most of Asia and Africa.

Since 1961, most opioids have been internationally controlled under the United Nations (UN) Single Convention on Narcotic Drugs, amended by the 1972 Protocol.^11^ Countries and territories that sign the treaty commit to implementing the drug control measures while ensuring access for medical and scientific purposes. The International Narcotics Control Board (INCB), an independent body of the UN, is responsible for monitoring implementation and compliance with international drug control treaties, which requires governments to report annual statistics on narcotic consumption relating to controlled drugs.^12^ When using data from the INCB, the term ‘consumption’ is used as per article 1, paragraph 2 of the Single Convention and 1972 Protocol; a drug is considered as ‘consumed’ when it has been supplied to any person or enterprise for retail distribution, medical use or scientific research.^13^ To date, most pharmacoepidemiological studies on opioid use have been conducted in high-income countries owing to data provisions.^14–20^ Therefore, the use of INCB data allows opioid consumption to be compared globally, regionally and nationally for countries in all income groups.

Several observational studies using data from the INCB have evaluated barriers to access and have shown complex interactions between historical, social, cultural, economic and political decisions, limiting medical access to opioids.^8,9,21–31^ However, most studies on controlled opioid consumption have focused on cancer pain and palliative care and have examined morphine alone or a subset of five opioids,^9,10,21,32^ which may underestimate the scale of the problem. To the best of our knowledge, no studies have evaluated controlled opioid consumption trends that simultaneously include opioids for anaesthesia, analgesia, the management of diarrhoea, opioid dependence and cough suppression. Therefore, this study aimed to determine global, regional and national consumption of all controlled opioids, where consumption refers to the total amount of opioids distributed for medical purposes and excludes recreational use.

## Methods

### Study design and data sources

We designed and conducted a cross-sectional observational study using data from the INCB.^33^ The INCB is an independent body of the UN, and it monitors the implementation of international drug control conventions, including the Single Convention on Narcotic Drugs of 1961, which requires governments to report annual statistics on narcotic consumption relating to controlled drugs.^12^ Consumption refers to the total amount of a narcotic distributed for medical purposes at the retail level (i.e. to institutions and programmes that are licensed to dispense to patients).^13^ We obtained data on consumption in kilograms from 2015 to 2017 and removed 26 non-opioid substances (e.g. cannabis, coca leaf and cocaine) to create a data set of all opioids consumed. The included and excluded substances are listed in Box S1 in the Supplement.

### Data analysis

We included all countries and non-metropolitan territories (n = 214) that provided data to the INCB. We converted raw consumption data from kg to mg and divided by global, regional and country-specific populations for 2016 to calculate mean rates of consumption (mg/person). The INCB recommends using a 3-year mean to display the data, and previous studies have used this to account for annual variations in reporting, providing more stable data.^23^ We determined the medians and interquartile ranges (IQR) for the consumption of controlled opioids worldwide. We categorized countries into deciles based on their consumption using the *xtile* function in Stata^34^ and calculated the percentage of opioids consumed and the percent of the population in each decile. We ranked countries by the rate of consumption (mg/person). We categorized opioids into their Anatomical Therapeutic Classification (ATC) index subgroups (i.e. analgesics, opioid substitution therapies, cough suppressants, anaesthetics and antidiarrheals) and ranked types of opioids by volume of consumption.

### Software and data sharing

We used Stata v16^35^ for all statistical analyses and pandas and plotly modules in Jupyter Notebooks with Python v3 for choropleth maps. The study materials, data and statistical code are all openly available on the Open Science Framework^36^ and GitHub.^37^ We used the STROBE reporting guidelines in writing our manuscript (see the Supplement for the completed checklist).

## Results

Globally, over 700 tonnes (710,043 kg) of controlled opioids were consumed in 2015–2017, an average of 32 mg/person each year. Regionally, the Americas had the greatest average consumption (144 mg/person), followed by Oceania (132 mg/person), Europe (98 mg/person), Asia (3.5 mg/person), and Africa (1.4 mg/person) (see Figure S1 in the Supplement).

**Figure 1.**
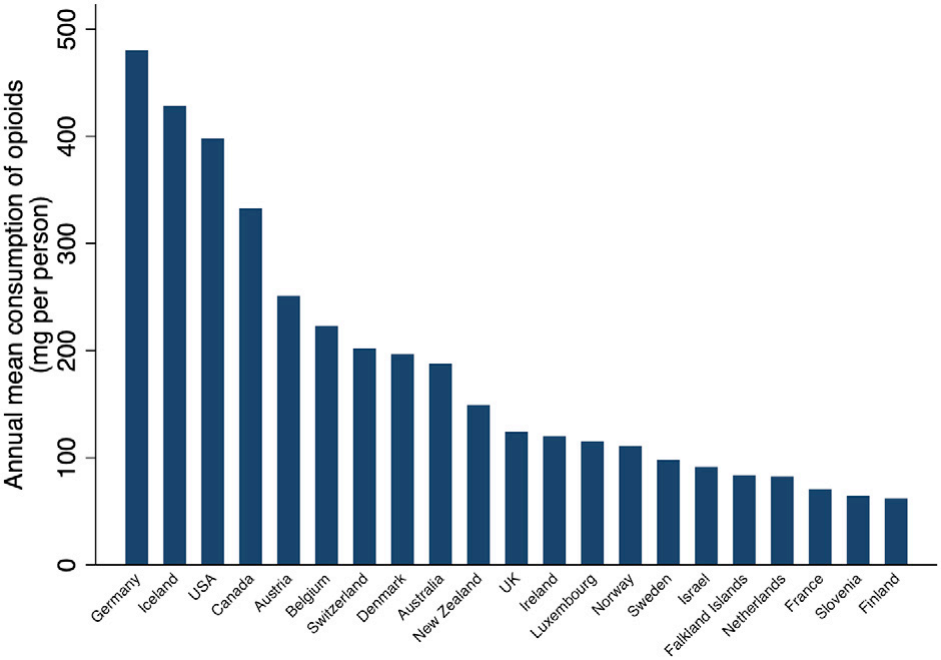
Countries (n = 21) in the top decile for controlled opioid consumption (mg per person) for 2015–2017. Countries in the top decile consumed 89% of controlled opioids between 2015 and 2017 and accounted for only 10% of the 2016 world population. Consumption refers to the total amount of controlled opioids distributed for medical purposes and excludes recreational use; it was calculated by determining the 3-year mean for 2015–2017 and dividing this by the 2016 population for each country. Data were obtained from the International Narcotics Control Board.

**Figure 2.**
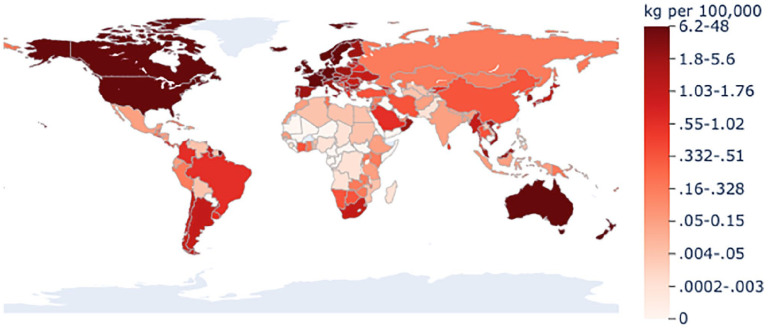
Annual mean consumption of controlled opioids for 2015–2017 grouped by deciles for all countries and non-metropolitan territories (n = 214). Consumption refers to the total amount of controlled opioids distributed for medical purposes and excludes recreational use; it was calculated by determining the 3-year mean for 2015–2017 and dividing this by the 2016 population for each country. Data were obtained from the International Narcotics Control Board.

Nationally, countries consumed a median of 3.3 mg/person (IQR = 0.24–14.8; range = 0–480 mg/person). Consumption of controlled opioids was the highest in Germany (480 mg/person), followed by Iceland (428 mg/person), the United States (398 mg/person), and Canada (333 mg/person) (see Figure 1 and Table S1 in the Supplement). There were 21 countries in the top decile of consumption; they consumed 89% (630 tonnes) of the world’s controlled opioids and accounted for only 9.7% of the world’s population (687 million of 7.1 billion people in 2016) (see Table S2 in the Supplement). Deciles that accounted for larger percentages of the population, decile 6 (24% of the population), decile 3 (21% of the population), and decile 4 (13% of the population), consumed only 2.7%, 0.28%, and 0.32% of the world’s controlled opioids, respectively (see Table S2 in the Supplement). Thirty-five countries, accounting for 3.1% of the population, reported no consumption of controlled opioids to the INCB. Figure 2 displays the distribution of controlled opioids consumed for all countries and territories by decile.

**Figure 3.**
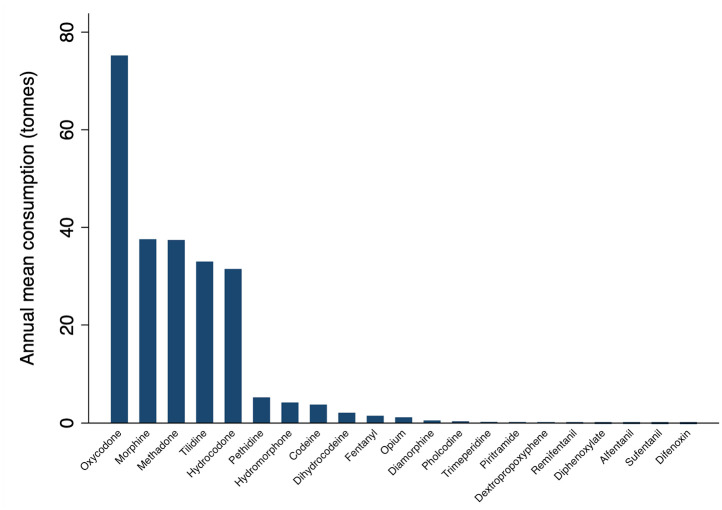
Annual mean consumption (tonnes) in 2015–2017 by type of opioid where consumption refers to the total amount of controlled opioids distributed for medical purposes and excludes recreational use in all countries and non-metropolitan territories (n = 214) with data provided by the International Narcotics Control Board.

Globally, oxycodone was the most heavily consumed, accounting for one-third of all opioids, followed by morphine (15.9%), methadone (15.8%), and tilidine (13.9%) (see Figure 3 and Table S3 in the Supplement). Analgesics (493 tonnes, n = 12 opioids) were the most common Anatomical Therapeutic Chemical (ATC) category of opioids consumed, followed by opioid substitution therapies (114 tonnes, n = 2 opioids), cough suppressants (95 tonnes, n = 2 opioids), anaesthetics (0.3 tonnes, n = 3 opioids), and antidiarrheals (0.07 tonnes, n = 2 opioids) (see Table S3 in the Supplement).

## Discussion

The global consumption of opioids was disproportionately distributed; 6.4 billion people worldwide had little or no access to controlled opioids for medical uses. Yet an average of 32 mg for every person in 2016 was available. Consumption per person in Oceania surpassed that in Europe, and the Americas consumed 41 times more than Asia and 103 times more than Africa. Nationally, consumption per head of population in Germany and Iceland exceeded that in North America. Analgesics were the most commonly consumed controlled opioids, with oxycodone, morphine and methadone leading the way.

Compared with previous research,^8^ there has been a shift in consumption from North American countries to Germany and Iceland. This shift may be due to mitigation strategies (e.g. prescription monitoring programmes and pill mill laws) in North America which are reducing medical access to opioids.^38–40^ However, this restriction has had devastating consequences. Opioid-dependent people without access to opioid substitution therapy, such as methadone, have moved to illicit sources of opioids, and unintended overdose deaths have rapidly increased.^41^ Lessons from the North American opioid crisis must be considered by all countries when designing strategies and clinical guidelines that improve access to opioids while limiting dependence and harm. There are currently no National Institute for Health and Care Excellence (NICE) guidelines for prescribing opioids in the United Kingdom, and the WHO has recently retracted its two main guidelines for opioid use because of interference by opioid manufacturers.^42,43^ Thus, a coordinated effort is needed across all countries to develop evidence-based guidelines that promote the appropriate use of opioids and warn against the significant harms of opioids, and support non-pharmacological approaches to managing chronic pain.

We found disparities and wide variations in consumption regionally and nationally. Previous research has highlighted many barriers to accessing opioids, including the absence of training, fear of dependence and diversion, problems in sourcing, the complexities of regulating opioids as internationally controlled substances, and cultural and social attitudes.^8,23–25^ In countries with low opioid consumption, there may be access to non-opioid alternatives, such as paracetamol for pain and ketamine or bupivacaine for anaesthesia, and varying national guidance that may impact the use of controlled opioids. The WHO and the INCB have made efforts to encourage governments to have balanced policies and health systems that ensure opioids are both available and accessible to those in need. However, our findings question whether much has been achieved to overcome the barriers, as disparities in access to opioids persist.

While access to opioids for acute pain, palliative care and cancer pain should continue to be advocated, there is growing concern that pharmaceutical companies have started US-style marketing techniques in low- and middle-income countries.^44,45^ Prescribers in these countries must therefore be prepared to deal with marketing campaigns to prescribe and use opioids and be educated about the consequences of conflicts of interests until sanctions can be enforced to prevent a North American opioid crisis.

### Strengths and limitations

We included data from all countries and non-metropolitan territories for all available types of opioids (anaesthetics, analgesics, antidiarrheals, cough suppressants and opioid substitution therapies). Thus, our findings are representative of the global population between 2015 and 2017. We standardized consumption using 2016 population statistics to create a rate per person, which assumes that opioids were consumed by individuals of all ages, including children. However, it is not possible to elucidate what proportion of people consumed or received opioids in each country. Data for opioids that are not regulated as internationally controlled substances (e.g. tramadol and buprenorphine) are not reported to the INCB. Thus, this analysis represents the consumption of internationally controlled opioids. However, we have included 225 substances, which incorporate all types of available opioids rather than focusing on analgesics or a subset of opioids, as previous researchers have done.^9,10,21,32^ We found 35 countries that reported no consumption of opioids to the INCB, and it is unclear whether this reflects actual consumption, as data may be late, unreported or submitted inaccurately, as previously described.^8,10^ We categorized opioids using the ATC index; however, there are opioids, such as fentanyl, that may be used as analgesics and anaesthetics, which were not accounted for.

The effects of opioids vary by type and weight (e.g. 10 mg of morphine will have a greater effect than 10 mg of codeine), which morphine equivalent conversion would account for if accurate conversion were possible. We measured consumption using weight in mg adjusted for country population, as potency ratios and defined daily dose (DDD) conversion factors are not available for all opioid substances included in our analysis. The WHO created DDDs, a technical measure given to most medicines with an ATC code, defined as ‘the assumed average maintenance dose per day for a drug used for its main indication in adults’.^46^ A DDD provides an estimate of consumption rather than the actual use of a drug, and its use in opioid research is discouraged.^47^ Thus, our findings may not be easily comparable with those of most previous studies,^7,8,31^ in which consumption has been measured using oral morphine equivalents or DDDs.

## Conclusion

The consumption of controlled opioids remains low in most of the world while being extremely high in a few countries. Countries with very high rates of consumption may need to implement measures that promote appropriate prescribing. Governments and international bodies should work together to update and develop clinical guidelines, policies, and health systems that promote access to opioids for acute pain, cancer pain, palliative care, anaesthesia and opioid dependence, assess the evidence base of opioids as antidiarrheals and cough suppressants, implement strategies to reduce the harms of opioids, and mitigate the outcomes that arise when pharmaceutical companies act only in their own interests and not those of patients.

## Supplemental Material

sj-docx-1-bjp-10.1177_20494637211013052 – Supplemental material for Global, regional, and national consumption of controlled opioids: a cross-sectional study of 214 countries and non-metropolitan territories Click here for additional data file. Supplemental material, sj-docx-1-bjp-10.1177_20494637211013052 for Global, regional, and national consumption of controlled opioids: a cross-sectional study of 214 countries and non-metropolitan territories by Georgia C Richards, Jeffrey K Aronson, Kamal R Mahtani and Carl Heneghan in British Journal of Pain

sj-docx-2-bjp-10.1177_20494637211013052 – Supplemental material for Global, regional, and national consumption of controlled opioids: a cross-sectional study of 214 countries and non-metropolitan territories Click here for additional data file. Supplemental material, sj-docx-2-bjp-10.1177_20494637211013052 for Global, regional, and national consumption of controlled opioids: a cross-sectional study of 214 countries and non-metropolitan territories by Georgia C Richards, Jeffrey K Aronson, Kamal R Mahtani and Carl Heneghan in British Journal of Pain
