# Supplementary material for: Global, regional, and national consumption of controlled opioids: a cross-sectional study of 214 countries and non-metropolitan territories
Source: Br J Pain. 2021 May 4;16(1):34–40. doi: 10.1177/20494637211013052 (PMC8801686; doi:10.1177/20494637211013052)
Supplement: sj-docx-1-bjp-10.1177_20494637211013052 – Supplemental material for Global, regional, and national consumption of controlled opioids: a cross-sectional study of 214 countries and non-metropolitan territories [file sj-docx-1-bjp-10.1177_20494637211013052.docx]

**Supplement: Additional tables and figures**

**Box S1:** Substances included and excluded from the International Narcotic Control Board data on narcotic consumption, in alphabetical order.

Opioids **included** in our opioid consumption dataset:

1. (+)-cis-3-methylfental
2. 3-Acetylmorphine
3. 3-Methylfentanyl
4. 3-Methylthiofentanyl
5. 3-Monoacetylmorphine
6. 4-Fluoroisobutyrfentanyl
7. 6-Acetylmorphine
8. 6-Monoacetylmorphine
9. Acetorphine
10. Acetyl-alpha-methylfentanyl
11. Acetyldihydrocodeine
12. Acetylfentanyl
13. Acetylmethadol
14. Acetylmorphine
15. Acrylfentanyl
16. AH-7921
17. Alfentanil
18. Allylprodine
19. Alphacetylmethadol
20. Alphameprodine
21. Alphamethadol
22. alpha-Methylfentanyl
23. alpha-Methylthiofentanyl
24. Alphaprodine
25. Anileridine
26. Benzethidine
27. Benzoylmorphine
28. Benzylmorphine
29. Betacetylmethadol
30. beta-Hydroxy-3-methyl fentanyl
31. beta-Hydroxyfentanyl
32. Betameprodine
33. Betamethadol
34. Betaprodine
35. Bezitramide
36. Butyrfentanyl
37. Carfentanil
38. Carfentanyl
39. Clonitazene
40. Codeine
41. Codeine-6GLUC
42. Codeine-6-glucuronide
43. Codeine-Methyl
44. Codeine-N-oxide
45. Codoxime
46. Conc. of poppy straw (C) ACA
47. Conc. of poppy straw (C) AMA
48. Conc. of poppy straw (C) AOA
49. Conc. of poppy straw (C) ATA
50. Conc. of poppy straw (C) GW
51. Conc. of poppy straw (M) ACA
52. Conc. of poppy straw (M) AMA
53. Conc. of poppy straw (M) AOA
54. Conc. of poppy straw (M) ATA
55. Conc. of poppy straw (M) GW
56. Conc. of poppy straw (N) GW
57. Conc. of poppy straw (O)
58. Conc. of poppy straw (O) ACA
59. Conc. of poppy straw (O) AMA
60. Conc. of poppy straw (O) AOA
61. Conc. of poppy straw (O) ATA
62. Conc. of poppy straw (O) GW
63. Conc. of poppy straw (O)-AOA
64. Conc. of poppy straw (T)
65. Conc. of poppy straw (T) ACA
66. Conc. of poppy straw (T) AMA
67. Conc. of poppy straw (T) AOA
68. Conc. of poppy straw (T) ATA
69. Conc. of poppy straw (T) GW
70. Conc. of poppy straw (T)-ATA
71. Conc. of poppy straw (total) anhydrous codeine alkaloid
72. Conc. of poppy straw (total) anhydrous morphine alkaloid
73. Conc. of poppy straw (total) anhydrous oripavine alkaloid
74. Conc. of poppy straw (total) anhydrous thebaine alkaloid
75. Concentrate of poppy straw (M)
76. Concentrate of poppy straw (M)AMA
77. Concentrate of poppy straw (M)-ATA
78. Desomorphine
79. Dextromoramide
80. Dextropropoxyphene
81. Diampromide
82. Diethylthiambutene
83. Difenoxin
84. Dihydrocodeine
85. Dihydroetorphine
86. Dihydroisomorphin-6GLUC
87. Dihydromorphine
88. Dihydromorphine-6GLUC
89. Dihydrothebaine
90. Dimenoxadol
91. Dimepheptanol
92. Dimethylmorphine
93. Dimethylthiambutene
94. Dioxaphetyl butyrate
95. Diphenoxylate
96. Dipipanone
97. D-Isomethadone
98. Drotebanol
99. Ethylmethylthiambutene
100. Ethylmorphine
101. Etonitazene
102. Etorphine
103. Etorphine-3metheth
104. Etoxeridine
105. Fentanyl
106. Furanylfentanyl
107. Furethidine
108. Heroin
109. Hydrocodone
110. Hydromorphinol
111. Hydromorphone
112. Hydromorphone-3GLUC
113. Hydromorphone-N-oxide
114. Hydroxypethidine
115. Isomethadone
116. Ketobemidone
117. L-Alphacetylmethadol
118. Levo-A-acetylmethadol
119. Levomethorphan
120. Levomoramide
121. Levophenacylmorphan
122. Levopropoxyphene
123. Levorphanol
124. L-Isomethadone
125. L-Methadol
126. L-methadone
127. Metazocine
128. Methadone
129. Methadone intermediate
130. Methyldesorphine
131. Methyldihydromorphine
132. Metopon
133. Monoacetylmorphine
134. Moramide intermediate
135. Morpheridine
136. Morphine
137. Morphine-3,6DGLUC
138. Morphine-3BD,GLUC
139. Morphine-3-B-D-glucuronide
140. Morphine-3GLUC
141. Morphine-3-PROP
142. Morphine-6BD,GLUC
143. Morphine-6-B-D-glucuronide
144. Morphine-6GLUC
145. Morphine-DIMETETH
146. Morphine-METHYBRO
147. Morphine-METHYIOD
148. Morphine-N-oxide
149. MPPP
150. MT-45
151. Myrophine
152. Nicocodine
153. Nicodicodine
154. Nicomorphine
155. Noracymethadol
156. Norcodeine
157. Norlevorphanol
158. Normethadone
159. Normethadone intermediate
160. Normorphine
161. Normorphine-3GLUC
162. Norpipanone
163. Ocfentanyl
164. OLD Morphine-6GLUC
165. Opium
166. Opium - non medical use
167. Opium marc
168. Opium, prepared
169. Oripavine
170. Oxycodone
171. Oxycodone-N-oxide
172. Oxymorphone
173. Papaver bracteatum
174. para-Fluorofentanyl
175. PEPAP
176. Pethidine
177. Pethidine intermediate A
178. Pethidine intermediate B
179. Pethidine intermediate C
180. Phenadoxone
181. Phenampromide
182. Phenazocine
183. Phenomorphan
184. Phenoperidine
185. Pholcodine
186. Piminodine
187. Piritramide
188. Poppy straw (C) GW
189. Poppy straw (M)
190. Poppy straw (M) GW
191. Poppy straw (M) GW-ACA
192. Poppy straw (M) GW-AMA
193. Poppy straw (M) GW-AOA
194. Poppy straw (M) GW-ATA
195. Poppy straw (M)-ACA
196. Poppy straw (M)-AMA
197. Poppy straw (M)-AOA
198. Poppy straw (M)-ATA
199. Poppy straw (N) GW
200. Poppy straw (T)
201. Poppy straw (T) GW
202. Poppy straw (T) GW-ACA
203. Poppy straw (T) GW-AMA
204. Poppy straw (T) GW-AOA
205. Poppy straw (T)-ACA
206. Poppy straw (T)-AOA
207. Poppy straw (T)-ATA
208. Poppy straw (total) anhydrous codeine alkaloid
209. Poppy straw (total) anhydrous morphine alkaloid
210. Poppy straw (total) anhydrous thebaine alkaloid
211. Proheptazine
212. Properidine
213. Propiram
214. Racemethorphan
215. Racemoramide
216. Racemorphan
217. Remifentanil
218. Sufentanil
219. Tetrahydrofuranylfentanyl
220. Thebacon
221. Thebaine
222. Thiofentanyl
223. Tilidine
224. Trimeperidine
225. U-47700

Opioids **excluded** from our opioid consumption dataset:

1. Cannabis
2. Cannabis (non-medical use)
3. Cannabis oil
4. Cannabis resin
5. Cannabis resin-non medical use
6. Coca leaf
7. Coca leaf - non medical use
8. Coca paste
9. Cocaine
10. DUMMY
11. Ecgonine
12. Ecgonine-Benetest
13. Ecgonine-Bezest,4
14. Ecgonine-Bezprest
15. Ecgonine-Cinmeest
16. Ecgonine-Diflbene
17. Ecgonine-Ethylest
18. Ecgonine-Methyest
19. Ecgonine-M-Hydrox
20. Not covered substances
21. Other
22. Schedule III preparations
23. Special C.P.S
24. Unknown (ND019---)
25. Unspecified sources
26. Blank


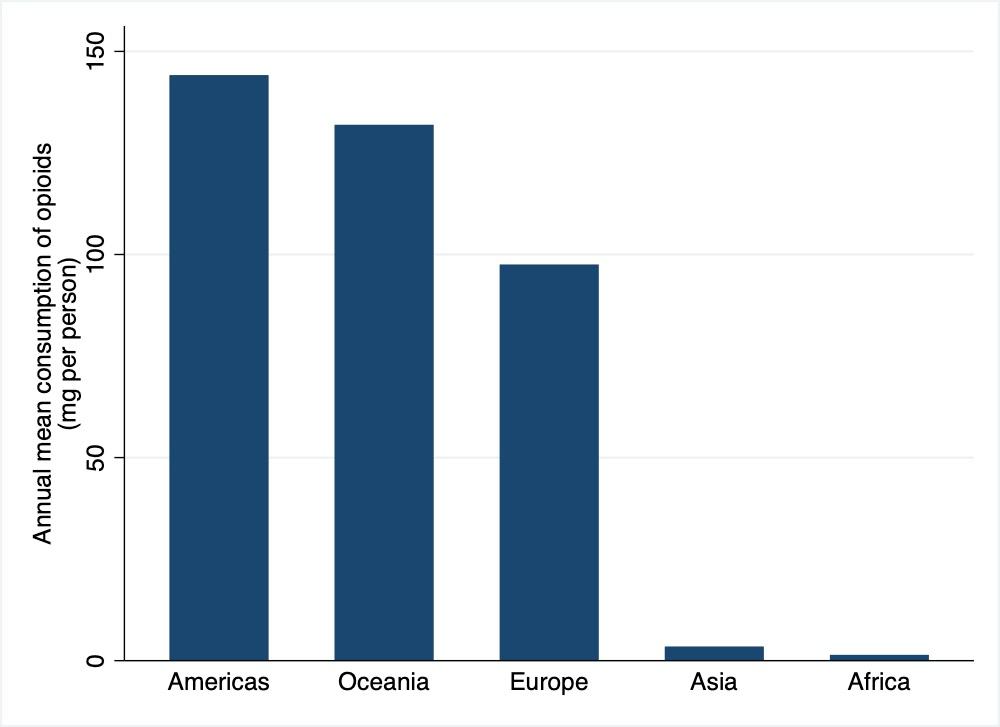


**Figure S1**: Annual mean consumption of controlled opioids (mg/person) regionally between 2015–2017. Consumption refers to the total amount of opioids distributed for medical purposes and excludes recreational use; it was calculated by determining the three year mean for 2015 to 2017 and dividing this by the 2016 population for each country. Data were obtained from the International Narcotics Control Board.

**Table S1:** Mean consumption of controlled opioids (mg/person) for each country, territory, and state (n=214) in 2015–2017. Countries are in descending order by consumption; countries who reported no consumption or no data to the International Narcotic Control Board are ordered alphabetically.

| **Country** | **Mean opioid consumption (mg/person)** |
| --- | --- |
| Germany | 480.28 |
| Iceland | 428.44 |
| USA | 397.85 |
| Canada | 332.67 |
| Austria | 251.01 |
| Belgium | 222.88 |
| Switzerland | 201.85 |
| Denmark | 196.65 |
| Australia | 187.83 |
| New Zealand | 149.34 |
| UK | 124.25 |
| Ireland | 119.98 |
| Luxembourg | 115.44 |
| Norway | 110.80 |
| Sweden | 97.93 |
| Israel | 91.50 |
| Falkland Islands | 83.76 |
| Netherlands | 82.39 |
| France | 70.82 |
| Slovenia | 64.45 |
| Finland | 61.84 |
| Mauritius | 55.53 |
| Italy | 55.17 |
| Spain | 50.88 |
| Wallis & Futuna Islands | 45.31 |
| Malta | 42.15 |
| Gibraltar | 39.89 |
| Norfolk Island | 37.23 |
| Sint Maarten | 32.13 |
| Barbados | 31.19 |
| North Macedonia | 26.08 |
| Estonia | 26.07 |
| Bulgaria | 25.53 |
| Cyprus | 25.44 |
| Christmas Island | 24.96 |
| Hong Kong | 24.17 |
| Czechia | 21.58 |
| Seychelles | 20.91 |
| Montserrat | 19.80 |
| Republic of Korea | 19.64 |
| Oman | 19.29 |
| Portugal | 17.93 |
| French Polynesia | 17.71 |
| Poland | 17.59 |
| Viet Nam | 17.48 |
| Andorra | 17.45 |
| New Caledonia | 16.53 |
| Malaysia | 16.41 |
| South Africa | 16.38 |
| Bahamas | 15.67 |
| Palau | 15.53 |
| Myanmar | 14.82 |
| Georgia | 14.79 |
| Croatia | 14.79 |
| Argentina | 14.33 |
| Slovakia | 14.23 |
| Chile | 12.54 |
| Bahrain | 12.06 |
| Latvia | 11.52 |
| Anguilla | 11.40 |
| Trinidad & Tobago | 11.09 |
| Japan | 10.82 |
| Romania | 10.46 |
| Ukraine | 10.25 |
| Guyana | 10.22 |
| Lithuania | 10.01 |
| Hungary | 9.95 |
| Serbia | 9.93 |
| Greece | 8.27 |
| Colombia | 7.66 |
| Uruguay | 7.42 |
| Jamaica | 7.24 |
| Costa Rica | 6.79 |
| Dominica | 6.79 |
| China, Macao SAR | 6.62 |
| Saudi Arabia | 6.36 |
| Turks & Caicos Islands | 6.34 |
| Sri Lanka | 6.00 |
| Niue | 6.00 |
| Kyrgyzstan | 5.94 |
| Saint Vincent & the Grenadines | 5.83 |
| Saint Helena | 5.73 |
| Brazil | 5.66 |
| Montenegro | 5.66 |
| Cook Islands | 5.61 |
| Moldova (the Republic of) | 5.54 |
| Albania | 5.06 |
| Belarus | 4.85 |
| Timor-Leste | 4.75 |
| El Salvador | 4.48 |
| British Virgin Islands | 4.48 |
| Democratic People's Republic of Korea | 4.48 |
| Eswatini | 4.34 |
| Belize | 4.33 |
| Lebanon | 4.12 |
| Tonga | 4.00 |
| Jordan | 3.98 |
| Bosnia & Herzegovina | 3.92 |
| Côte d'Ivoire | 3.90 |
| China | 3.85 |
| Namibia | 3.66 |
| Brunei Darussalam | 3.64 |
| Armenia | 3.64 |
| Curaçao | 3.48 |
| Kuwait | 3.43 |
| Islamic Republic of Iran | 3.33 |
| Turkey | 3.32 |
| Fiji | 3.28 |
| Thailand | 3.22 |
| Panama | 3.18 |
| Botswana | 3.17 |
| Singapore | 3.15 |
| Mongolia | 3.13 |
| Tunisia | 2.74 |
| Ghana | 2.67 |
| Zimbabwe | 2.41 |
| Federated States of Micronesia | 2.31 |
| Peru | 2.27 |
| Cuba | 2.21 |
| Uganda | 2.03 |
| Tuvalu | 2.03 |
| Kenya | 1.93 |
| Guatemala | 1.86 |
| Russian Federation | 1.81 |
| Papua New Guinea | 1.78 |
| Ascension Island | 1.65 |
| Syrian Arab Republic | 1.64 |
| Qatar | 1.61 |
| Zambia | 1.55 |
| United Arab Emirates | 1.53 |
| Malawi | 1.49 |
| Kazakhstan | 1.31 |
| Morocco | 1.29 |
| Senegal | 1.24 |
| Tanzania (United Republic of) | 1.16 |
| Dominican Republic | 1.02 |
| Solomon Islands | 0.91 |
| Ecuador | 0.86 |
| Bangladesh | 0.83 |
| Maldives | 0.76 |
| Azerbaijan | 0.74 |
| Honduras | 0.73 |
| Mexico | 0.71 |
| Indonesia | 0.71 |
| Nicaragua | 0.68 |
| Cabo Verde | 0.60 |
| Rwanda | 0.60 |
| Afghanistan | 0.56 |
| Ethiopia | 0.54 |
| India | 0.53 |
| Nepal | 0.52 |
| Lao People's Democratic Republic | 0.46 |
| Egypt | 0.38 |
| Philippines | 0.36 |
| Uzbekistan | 0.33 |
| Turkmenistan | 0.31 |
| Bolivia (Plurinational State of) | 0.30 |
| Suriname | 0.29 |
| Kiribati | 0.29 |
| Bhutan | 0.25 |
| Sudan | 0.24 |
| Benin | 0.24 |
| Mozambique | 0.22 |
| Algeria | 0.16 |
| Libya | 0.15 |
| Bolivarian Republic of Venezuela | 0.13 |
| Tajikistan | 0.11 |
| Togo | 0.11 |
| Comoros | 0.063 |
| Burkina Faso | 0.054 |
| Burundi | 0.049 |
| Madagascar | 0.037 |
| Pakistan | 0.033 |
| Haiti | 0.028 |
| Democratic Republic of the Congo | 0.026 |
| Nigeria | 0.012 |
| Chad | 0.006 |
| Sierra Leone | 0.005 |
| Angola | 0.002 |
| Antigua & Barbuda | 0 |
| Aruba | 0 |
| Bermuda | 0 |
| Cambodia | 0 |
| Cameroon | 0 |
| Cayman Islands | 0 |
| Central African Republic | 0 |
| Cocos (Keeling) Islands | 0 |
| Congo | 0 |
| Djibouti | 0 |
| Equatorial Guinea | 0 |
| Eritrea | 0 |
| Gabon | 0 |
| Gambia | 0 |
| Grenada | 0 |
| Guinea | 0 |
| Guinea-Bissau | 0 |
| Iraq | 0 |
| Lesotho | 0 |
| Liberia | 0 |
| Mali | 0 |
| Marshall Islands | 0 |
| Mauritania | 0 |
| Nauru | 0 |
| Niger | 0 |
| Paraguay | 0 |
| Saint Kitts & Nevis | 0 |
| Saint Lucia | 0 |
| Samoa | 0 |
| Sao Tome & Principe | 0 |
| Somalia | 0 |
| South Sudan | 0 |
| Tristan da Cunha | 0 |
| Vanuatu | 0 |
| Yemen | 0 |

**Table S2:** Deciles of opioid consumption for all countries, territories, states, and islands (n=214)

| **Decile** | **No. of countries** | **Range of consumption (mg/person)** | **% of consumption** | **% of population** |
| --- | --- | --- | --- | --- |
| 10 | 21 | 62–480 | 88.75 | 9.7 |
| 9 | 21 | 18–56 | 3.260 | 2.9 |
| 8 | 22 | 10.3–17.7 | 3.256 | 7.7 |
| 7 | 21 | 5.6–10.2 | 0.979 | 5.1 |
| 6 | 22 | 3.32–5.54 | 2.709 | 23.5 |
| 5 | 21 | 1.6–3.3 | 0.437 | 6.5 |
| 4 | 21 | 0.54–1.55 | 0.319 | 13.0 |
| 3 | 22 | 0.049–0.527 | 0.282 | 20.9 |
| 2 | 8 | 0.002–0.037 | 0.005 | 7.7 |
| 1 | 35 | 0–0 | – | 3.1 |

**Table S3**: Annual mean consumption of controlled opioids (kg) by type of opioid for all countries, territories, states, and islands (n=214) in descending order

| **Medicine** | **Total volume of consumption (kg)** |
| --- | --- |
| **Analgesics (n=12)** | **492,810** |
| Oxycodone | 225646 |
| Morphine | 112674 |
| Tilidine | 98929 |
| Pethidine | 15459 |
| Hydromorphone | 12603 |
| Codeine | 11318 |
| Dihydrocodeine | 6340 |
| Fentanyl | 4484 |
| Opium | 3505 |
| Trimeperidine | 677 |
| Piritramide | 609 |
| Dextropropoxyphene | 568 |
| **Opioid substitution therapies (n=2)** | **113,850** |
| Methadone | 112225 |
| Diamorphine | 1626 |
| **Cough suppressants (n=2)** | **95,486** |
| Hydrocodone | 94387 |
| Pholcodine | 1099 |
| **Anaesthetics (n=3)** | **300** |
| Remifentanil | 230 |
| Alfentanil | 58 |
| Sufentanil | 13 |
| **Antidiarrheal (n=2)** | **68** |
| Diphenoxylate | 65 |
| Difenoxin | 3.3 |

No consumption data were available for buprenorphine, butorphanol, dextromethorphan, eluxadoline, loperamide, nalbuphine, noscapine, papaveretum, pargeverine, pentazocine, tapentadol, or tramadol.

**References for Supplement**

[Bosetti, C., Santucci, C., Radrezza, S., Erthal, J., Berterame, S., & Corli, O. (2018). Trends in the consumption of opioids for the treatment of severe pain in Europe, 1990–2016. *Eur J Pain*](http://paperpile.com/b/ab5KT3/89dsu)2019; 23(4):697-707. <https://onlinelibrary.wiley.com/doi/abs/10.1002/ejp.1337>

[United Nations. (2019). *World Population Prospects 2019*. Department of Economic and Social Affairs, Population Division.](http://paperpile.com/b/ab5KT3/X7gpy) <https://population.un.org/wpp/Download/Standard/Population/>.

[WHO. (2019). *Population Data by country*. Global Health Observatory Data Repository; World Health Organization.](http://paperpile.com/b/ab5KT3/esmTS) <http://apps.who.int/gho/data/node.main.SDGPOP?lang=en>.
